# Size- and Surface- Dual Engineered Small Polyplexes for Efficiently Targeting Delivery of siRNA

**DOI:** 10.3390/molecules26113238

**Published:** 2021-05-27

**Authors:** Shuang Liu, Shaohui Deng, Xiaoxia Li, Du Cheng

**Affiliations:** 1 PCFM Lab of Ministry of Education & Guangzhou Key Laboratory of Flexible Electronic Materials and Wearable Devices, School of Materials Science and Engineering, Sun Yat-sen University, Guangzhou 510275, China; liush53@mail2.sysu.edu.cn (S.L.); lixiaoxia@mail.sysu.edu.cn (X.L.); 2 Zhongshan School of Medicine, Sun Yat-sen University, Guangzhou 510275, China

**Keywords:** small polyplex, pH-sensitive PEG shedding, disulfide bond-crosslinking, siRNA delivery, targeting delivery

## Abstract

Though siRNA-based therapy has achieved great progress, efficient siRNA delivery remains a challenge. Here, we synthesized a copolymer PAsp(-N=C-PEG)-PCys-PAsp(DETA) consisting of a poly(aspartate) block grafted with comb-like PEG side chains via a pH-sensitive imine bond (PAsp(-N=C-PEG) block), a poly(l-cysteine) block with a thiol group (PCys block), and a cationic poly(aspartate) block grafted with diethylenetriamine (PAsp(DETA) block). The cationic polymers efficiently complexed siRNA into polyplexes, showing a sandwich-like structure with a PAsp(-N=C-PEG) out-layer, a crosslinked PCys interlayer, and a complexing core of siRNA and PAsp(DETA). Low pH-triggered breakage of pH-sensitive imine bonds caused PEG shedding. The disulfide bond-crosslinking and pH-triggered PEG shedding synergistically decreased the polyplexes’ size from 75 nm to 26 nm. To neutralize excessive positive charges and introduce the targeting ligand, the polyplexes without a PEG layer were coated with an anionic copolymer modified with the targeting ligand lauric acid. The resulting polyplexes exhibited high transfection efficiency and lysosomal escape capacity. This study provides a promising strategy to engineer the size and surface of polyplexes, allowing long blood circulation and targeted delivery of siRNA.

## 1. Introduction

RNA interference (RNAi)-based therapy and its effector small interfering RNA (siRNA) have achieved great progress [1,2,3]. Though many siRNA-related publications (>100,000) and RNAi-based clinical trials (~60) have been evidenced, few (0.06%) of these studies cover the area of clinical therapeutics [4,5]. One of the major hurdles for siRNA clinical application is the lack of safe and effective delivery approaches [6]. Apart from local delivery of chemically modified naked siRNA, most systemic delivery methods of siRNA need the help of vectors to improve therapeutic efficacy by increasing siRNA accumulation in target tissue and reducing side effects caused by non-specific uptake [7]. The optimizations of size and surface properties are crucial factors to achieve extremely efficient siRNA delivery with minimal side effects [8].

The size of nanoparticles has significant impacts on their blood circulation half-life [9,10], tumor penetration [11,12], target cell binding [13], and cellular uptake [14]. For instance, nanoparticle size has to be larger than 10 nm to escape the renal filtration [15], and be smaller than 200 nm to avoid activation of the complementary system [16]. Not counting the effect of core composition and surface charge, in nonphagocytic cells, the maximum cellular uptake was observed when treated with nanoparticles with a size range of 10–60 nm [17,18,19]. When target cell-specific antibody was conjugated on the surface of nanoparticles, a diameter range from 25.4 to 30.2 nm showed the greatest cellular uptake [20]. Notably, nanoparticles with a size of 20 nm or less exhibited the greatest tumor penetration to kill tumor cells that are located in the deep tumor tissue and, thus, can survive chemotherapy [8,21]. Thus, a diameter range of 20–30 nm may be an optimal size to achieve excellent drug delivery efficiency. However, existing siRNA delivery systems mostly have a larger diameter out of range of 20–30 nm, which may result in low delivery efficiency. Therefore, the development of a size engineering approach to produce small nanoparticles for siRNA delivery is desirable.

Nanoparticles’ surface properties constitute another crucial factor to determine their delivery efficiency [22]. Though the positive surface charges on the nanoparticles favors the cellular uptake via strengthened interaction between negatively charged cell membranes and nanoparticles, they also cause system toxicity including hemolysis and platelet aggregation, resulting in rapid clearance from blood [23]. By contrast, the nanoparticles with a negative charge surface show longer circulation half-life than those with positive charge, while the longest blood half-life is observed using nanoparticles with neutral surface [24]. To prolong the blood circulation time of nanoparticles by avoiding opsonization, it is a common approach to form a hydrophilic out-layer by introducing PEG chains on the surface of nanoparticles [25,26]. Compared to terminal linear PEG, the branched and comb-like terminal ones showed higher circulation half-life and serum stability [27]. Besides, the targeting ligands, including small molecules (e.g., folate), peptides (e.g., iRGD), and antibody fragments (e.g., Her2 antibody), have been introduced to the surface of nanoparticles to enhance targeting delivery efficiency [28,29,30,31]. Thus, the surface properties of nanoparticles play important roles in achieving excellent siRNA delivery efficiency. To engineer the surface of nanoparticles, several approaches have been developed. The di- or tri-block copolymer containing the PEG block and cationic block were prepared and used in a complex with siRNA in order to form nanoparticles, with/without targeting ligands conjugated to PEG [32,33,34]. However, these approaches take both time and effort. An alternative approach was developed to facilitate surface engineering. The targeting ligand modified PEG was added to the surface of siRNA assemblies with cationic polymer (e.g., PEI) using a hierarchical assembly strategy [35,36].

**Figure 1:**
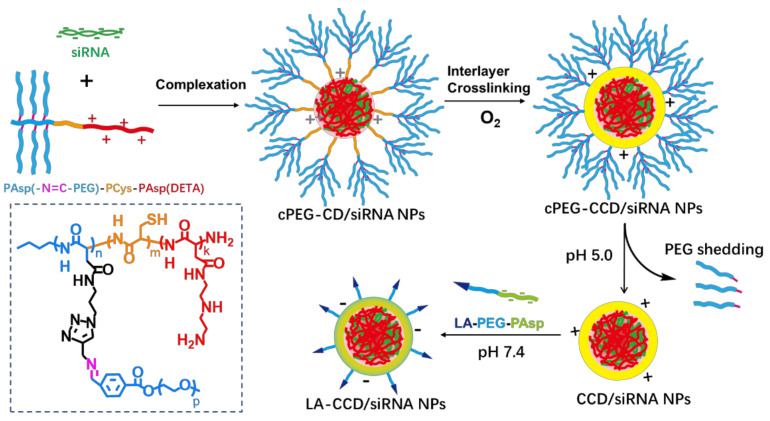
Schematic of the preparation of polyplex. The cationic copolymers cPEG-CD (i.e., PAsp(-N=C-PEG)-PCys-PAsp(DETA)) were used to complex with siRNA to form polyplexes cPEG-CD/siRNA nanoparticles (NPs). After O_2_ blowing, thiol groups in the PCys interlayer crosslinked. Under pH 5.0 condition, PEG dropped from polyplexes because of the breakage of pH-sensitive imide bond. Finally, the negatively charged copolymers LA-PEG-PAsp were used to coat the CCD/siRNA NPs (crosslinked CD/siRNA NPs), introducing the targeting ligand lauric acid and neutralizing excessive positive charges.

Consequently, both the size and surface engineering are necessary to ensure the long blood circulation half-life and highly efficient targeting delivery. However, few siRNA delivery systems integrated these features into one nanosystem. Here, we firstly prepared copolymer PAsp(-N=C-PEG)-PCys-PAsp(DETA) consisting of a poly(aspartate) block grafted with comb-like PEG side chains via a pH-sensitive imine bond, a poly(L-cysteine) block with a thiol group, and a cationic poly(aspartate) block grafted with diethylenetriamine (DETA). After PAsp(DETA) blocks were complexed with siRNA, the crosslinking by thiol groups in the PCys interlay reduced the nanoparticle size. In the acid condition, the breakage of the imine bond caused PEG shedding from nanoparticles, resulting in a further reduction in nanoparticle size. Finally, negatively charged copolymers with a targeting ligand lauric acid were hierarchically coated above positively charged nanoparticles, allowing long circulation and targeted delivery of siRNA (Figure 1).

## 2. Results and Discussion

### 2.1. Synthesis and Characterization of the PAsp(-N=C-PEG)-PCys-PAsp(DETA)

**Figure 2:**
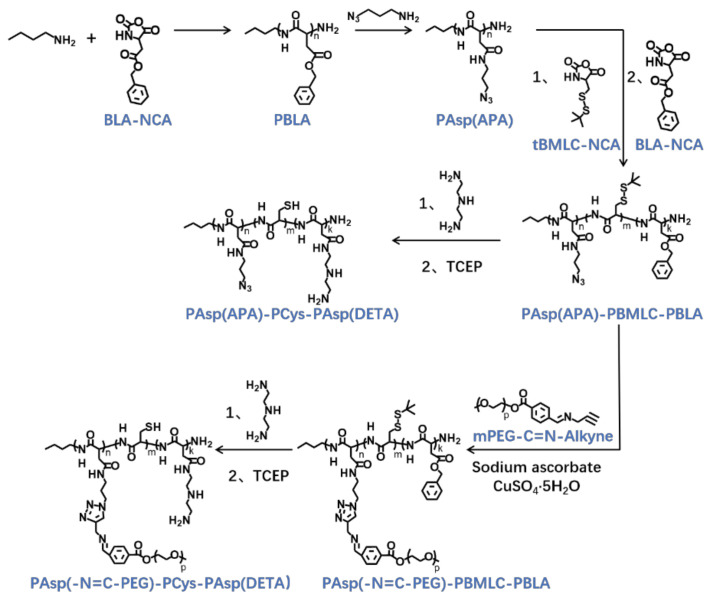
Synthetic route of PAsp(-N=C-PEG)-PCys-PAsp(DETA) and PAsp(APA)-PCys-PAsp(DETA).

The triblock cationic copolymer with a PEG lateral chain PAsp(-N=C-PEG)-PCys-PAsp(DETA), referred to here as cPEG-CD, was synthesized through ring-opening polymerization of BLA-NCA and tBMLC-NCA to form the main chain, through aminolysis reaction of DETA to form the cationic block PAsp(DETA), through reduction reaction of disulfide bond breakage to form the block with thiol groups, and through aminolysis reaction of 3-azidopropylamine and the azide–alkyne click reaction with mPEG-C=N-Alkyne to form the block grafted with comb-like PEG side chains via a pH-sensitive imine bond (Figure 2).

**Figure 3:**
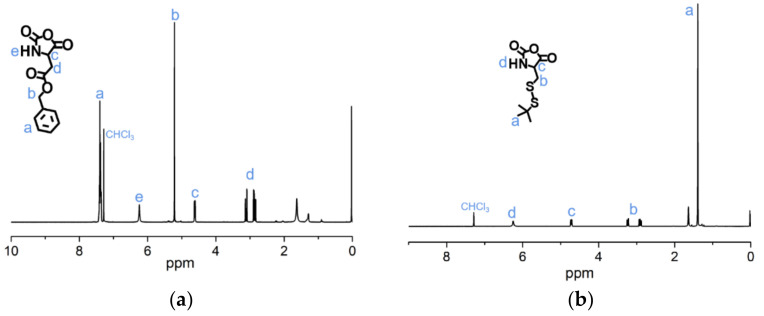
^1^H-NMR analyses of (**a**) BLA-NCA and (**b**) tBMLC-NCA in CDCl_3_.

The syntheses of BLA-NCA and tBMLC-NCA were performed according to previous reports [37,38]. The ^1^H-NMR analysis at 2.9 (m, 1H, -CH_2_-), 3.1 (m, 1H, -CH_2_-), 4.71 (dd, 1H, -CH-), 5.2 (s, 2H, -CH_2_-), 6.3 (s, 1H, -NH-) and 7.3 (s, 5H, ***Ar***-) ppm confirmed the successful synthesis of BLA-NCA (Figure 3a). The ^1^H-NMR analysis at 1.36 ppm (d, 9H, (CH_3_)_3_C-), 2.8 ppm (m, 1H, -CH_2_-), 3.23 ppm (m, 1H, -CH_2_-), 4.71 ppm (dd, 1H, -CH-), and 6.57 ppm (s, 1H, -NH-) confirmed the successful synthesis of tBMLC-NCA (Figure 3b).

**Figure 4:**
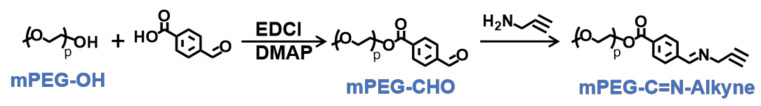
Synthetic route of mPEG-C=N-Alkyne.

**Figure 5:**
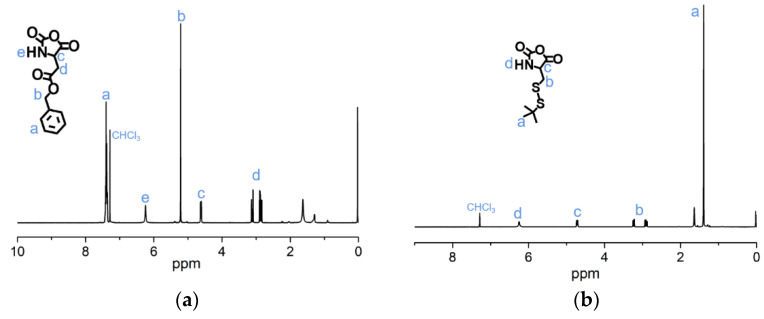
^1^H-NMR analyses of (**a**) mPEG-CHO and (**b**) mPEG-C=N-Alkyne in CDCl_3_.

To synthesize of pH-sensitive polymer mPEG-C=N-Alkyne, the mPEG-CHO was firstly prepared via esterification between mPEG-OH and 4-carboxybenzaldehyde, followed by a Schiff base reaction between mPEG-CHO and propargylamine (Figure 4). The ^l^H-NMR peaks (Figure 5a) at 3.40 ppm (s, C***H***_3_-), 3.65 ppm (m, -O(C***H***_2_)_2_O-), 4.35 ppm (t, -C***H***_2_OOC-), 7.90 ppm (dd, -***Ar***-), 8.15 ppm (dd, -***Ar***-) and 10.05 ppm (-C***H***O), and the ^l^H-NMR peaks (Figure 5b) at 2.60 ppm (s, -C≡C***H***), 3.40 ppm (s, C***H***_3_-), 3.65 ppm (m, -O(C***H***_2_)_2_O-), 4.35 ppm (t, -C***H***_2_OOC-), 4.50 ppm (s, -CH=N-C***H***_2_-), 7.90 ppm (dd, -***Ar***-), 8.15 ppm (dd, -***Ar***-) and 8.90 ppm (-C***H***=N-CH_2_-), verified the successful syntheses of mPEG-CHO and mPEG-C=N-Alkyne.

**Figure 6:**
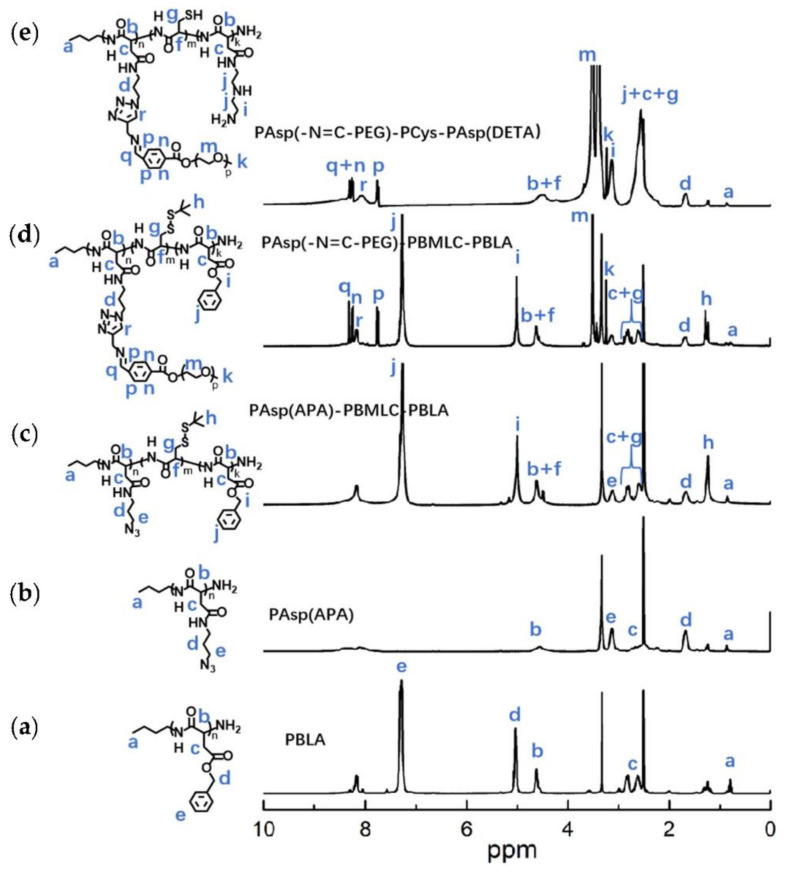
^1^H-NMR analyses of (**a**) PBLA, (**b**) PAsp(APA), (**c**) PAsp(APA)-PBMLC-PBLA, (**d**) PAsp(-N=C-PEG)-PBMLC-PBLA and (**e**) PAsp(-N=C-PEG)-PCys-PAsp(DETA) in DMSO-*d6*.

**Figure 7:**
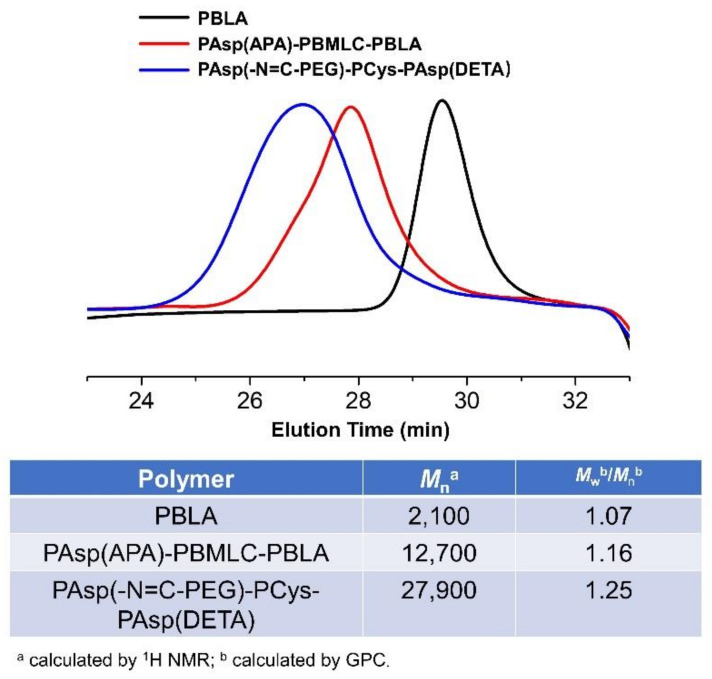
GPC curves, *M_n_* and molecular weight distributions of PAsp(-N=C-PEG)-PCys-PAsp(DETA), PAsp(APA)-PBMLC-PBLA, and PBLA.

To synthesize polymer PBLA, the BLA-NCA monomer was used to perform ring-opening polymerization using n-butylamine as an initiator (Figure 2). The ^1^H-NMR analysis at 0.85 ppm (t, C***H***_3_-), 2.6–2.9 ppm (d, -C***H***_2_COOCH_2_-), 4.80 ppm (s, -C***H***CH_2_COO-), 5.0 ppm (s, -C***H***_2_C_6_H_5_) and 7.30 ppm (m, C_6_***H***_5_CH_2_-) confirmed the successful synthesis of PBLA (Figure 6a). The degree of polymerization for PBLA was 10 and the number-average molecular weight (*M_n_*) was 2.1 kDa, as calculated based on the ^1^H-NMR spectrum. The GPC curve of PBLA showed a unimodal eluogram (Figure 7). The weight-average molecular weight/number-average molecular weight (*M*_w_/*M*_n_) of PBLA was 1.07. These results suggested the successful synthesis of PBLA.

**Figure 8:**
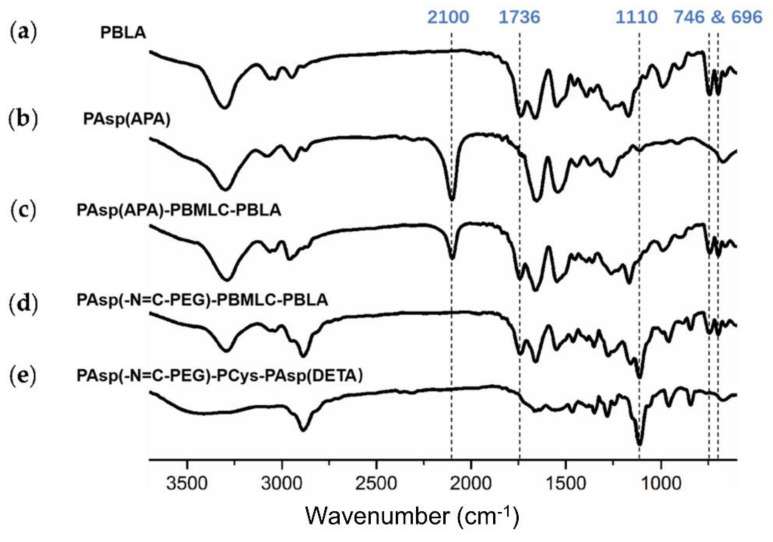
FTIR spectra of (**a**) PBLA, (**b**) PAsp(APA), (**c**) PAsp(APA)-PBMLC-PBLA, (**d**) Asp(-N=C-PEG)-PBMLC-PBLA and (**e**) PAsp(-N=C-PEG)-PCys-PAsp(DETA).

Next, poly(3-azidopropylamine) aspartamide, referred to here as PAsp(APA), was synthesized by ammonolysis reaction between PBLA and 3-azidopropylamine (Figure 2). The ^1^H-NMR analysis at 0.85 ppm (t, C***H***_3_-),1.65 ppm (m, N_3_CH_2_C***H***_2_-), 2.6–2.9 ppm (d, -CHC***H***_2_CONH-), 3.0 ppm (s, N_3_C***H***_2_-) and 4.80 ppm (s, -C***H***CH_2_CONH-) confirmed the successful synthesis of PAsp(APA) (Figure 6b). In the Fourier transform infrared (FTIR) spectrum of PAsp(APA), the characteristic peaks at 1736 cm^−1^ (*v*_C=O_, benzyl ester), and 746 and 696 cm^−1^ (*δ*_Ar-H_, benzenoid), of PBLA disappeared, and, at the same time, the characteristic peaks at 2100 cm^−1^ (*v*_-N3_, azide group) appeared, suggesting the successful deprotection of PBLA by ammonolysis with 3-azidopropylamine (Figure 8).

Subsequently, the triblock polymer PAsp(APA)-PBMLC-PBLA was synthesized through ring-opening polymerization of BLA-NCA and tBMLC-NCA using PAsp(APA) as an initiator (Figure 2). The ^1^H-NMR analysis at 0.85 ppm (t, C***H***_3_-), 1.3 ppm (s, -SS-C(C***H***_3_)_3_), 1.65 ppm (m, N_3_CH_2_C***H***_2_-), 2.6–2.9 ppm (d, -CHC***H***_2_CO-), 3.0 ppm (s, N_3_C***H***_2_-), 4.80 ppm (s, -C***H***CH_2_CO- and -C***H***CH_2_SS-), 5.0 ppm (s, -C***H***_2_C_6_H_5_) and 7.30 ppm (m, C_6_***H***_5_CH_2_-) confirmed the successful synthesis of PAsp(APA)-PBMLC-PBLA (Figure 6c). The degree of polymerization for PBMLC and PBLA were 7 and 45, respectively, as calculated based on the ^1^H-NMR spectrum. The *M_n_* and *M*_w_/*M*_n_ of PAsp(APA)-PBMLC-PBLA were 12.7 kDa and 1.16, respectively. The GPC curve of PAsp(APA)-PBMLC-PBLA showed a unimodal eluogram (Figure 7). The appearance of the characteristic peaks at 1736 cm^−1^ (*v*_C=O_, benzyl ester), and 746 and 696 cm^−1^ (δ_Ar-H_, benzenoid), indicated the successful synthesis of PAsp(APA)-PBMLC-PBLA (Figure 8c), consistent with the ^1^H-NMR spectrum results.

To prepare PAsp(-N=C-PEG)-PBMLC-PBLA, the resulting PAsp(APA)-PBMLC-PBLA was grafted with mPEG-C=N-Alkyne via a click reaction between the azide and alkynyl groups (Figure 2). The ^1^H-NMR analysis at 0.85 ppm (t, C***H***_3_-), 1.3 ppm (s, -SS-C(C***H***_3_)_3_), 1.65 ppm (m, -CH_2_C***H***_2_CH_2_NHCO-), 2.6–2.9 ppm (d, -CHC***H***_2_CO-), 3.40 ppm (s, C***H***_3_-), 3.65 ppm (m, -O(C***H***_2_)_2_O-), 4.80 ppm (s, -C***H***CH_2_CO- and -C***H***CH_2_SS-), 5.0 ppm (s, -C***H***_2_C_6_H_5_), 7.30 ppm (m, C_6_***H***_5_CH_2_-), 7.85 ppm (dd, -***Ar***-), 8.25 ppm (dd, -***Ar***-) and 8.35 ppm (-C***H***=N -) confirmed the successful synthesis of PAsp(-N=C-PEG)-PBMLC-PBLA (Figure 6d). In the FTIR spectrum of PAsp(-N=C-PEG)-PBMLC-PBLA, the disappearance of the characteristic peak at 2100 cm^−1^ (*v*_-N3_, azide group) and the appearance of the characteristic peak at 1110 cm^−1^ (*v*_C-O-C_, ether) suggested the conjugation of mPEG-C=N-Alkyne to the main chain of PAsp-PBMLC-PBLA (Figure 8d).

Finally, the target polymer PAsp(-N=C-PEG)-PCys-PAsp(DETA), referred to here as cPEG-CD, was synthesized through a ammonolysis reaction between PAsp(-N=C-PEG)-PBMLC-PBLA and diethylenetriamine (Figure 2). The ^1^H-NMR analysis at 0.85 ppm (t, C***H***_3_-), 1.65 ppm (m, -CH_2_C***H***_2_CH_2_NHCO-), 2.6–2.9 ppm (d, -CHC***H***_2_CO-, -CHC***H***_2_SH and -C***H***_2_NHC***H***_2_-), 3.2 ppm (s, NH_2_C***H***_2_-), 3.40 ppm (s, C***H***_3_-), 3.65 ppm (m, -O(C***H***_2_)_2_O-), 4.80 ppm (s, -C***H***CH_2_CO- and -C***H***CH_2_SH), 7.85 ppm (dd, -***Ar***-), 8.25 ppm (dd, -***Ar***-) and 8.35 ppm (-C***H***=N-) confirmed the successful synthesis of PAsp(-N=C-PEG)-PCys-PAsp(DETA) (Figure 6e). The *M_n_* and *M*_w_/*M*_n_ of PAsp(-N=C-PEG)-PCys-PAsp(DETA) were 27.9 kDa and 1.25, respectively. The intermolecular cross-linking between diethylenetriamine and PBLA hardly formed when a 20-fold molar excess of diethylenetriamine was reacted with the PBLA block [39,40], which was proven by the unimodal molecular weight distribution of PAsp(-N=C-PEG)-PCys-PAsp(DETA) (Figure 7). The FTIR spectrum analysis indicated the complete ammonolysis of PBLA (Figure 8e), which was consistent with the ^1^H-NMR spectrum results. Besides, the triblock cationic copolymer without a PEG lateral chain PAsp(APA)-PCys-PAsp(DETA), referred to here as nonPEG-CD, was prepared using a similar protocol and used as a control polymer in subsequent experiments.

### 2.2. Synthesis and Characterization of the Anionic Copolymer LA-PEG-PAsp

**Figure 9:**
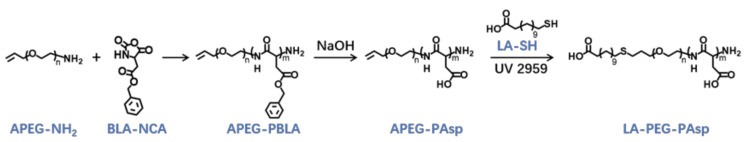
Synthetic route of LA-PEG-PAsp and APEG-PAsp.

The lauric acid-terminated anionic copolymer LA-PEG-PAsp was synthesized through polymerization of BLA-NCA with α-Allyl-ω-amino PEG (APEG-NH_2_) as an initiator, followed by hydrolysis of NaOH to generate the anionic polymer, and the thiol-ene click reaction to conjugate lauric acid (Figure 9).

**Figure 10:**
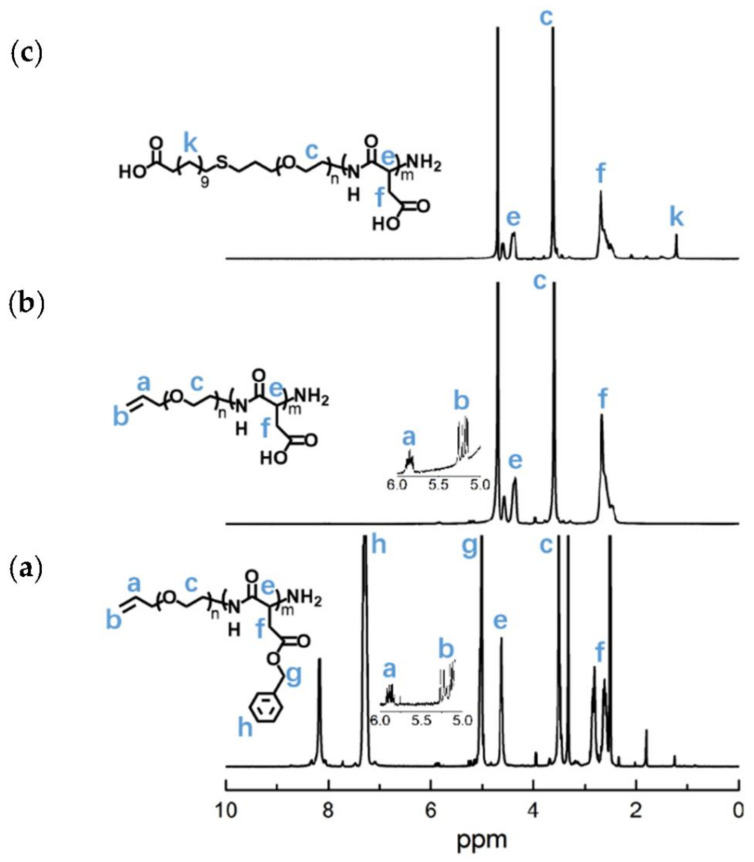
^1^H-NMR analyses of (**a**)APEG-PBLA, (**b**) APEG-PAsp, and (**c**) LA-PEG-PAsp.

The APEG_2.4K_-NH_2_ was used as an initiator to initiate the ring-opening polymeration of BLA-NCA in order to prepare APEG-PBLA. The ^1^H-NMR analysis at 2.6–2.9 ppm (d, -C***H***_2_COOCH_2_-), 3.56 ppm (s, -OC***H***_2_C***H***_2_O-), 4.80 ppm (s, -C***H***CH_2_COO-), 5.0 ppm (s, -C***H***_2_C_6_H_5_), 5.1–5.4 ppm (m, C***H***_2_ = CH-), 5.88 ppm (m, CH_2_ = C***H***-) and 7.30 ppm (m, C_6_***H***_5_CH_2_-) confirmed the successful synthesis of APEG-PBLA (Figure 10a). The degree of polymerization for PBLA was 80, as calculated based on the ^1^H-NMR spectrum. The *M_n_* of APEG-PBLA were 18.8 kDa. Then, the anionic polymer APEG-PAsp was synthesized through the hydrolysis of NaOH (Figure 9). The ^1^H-NMR analysis at 2.6–2.9 ppm (d, -C***H***_2_COOH), 3.56 ppm (s, -OC***H***_2_C***H***_2_O-), 4.60 ppm (s, -C***H***CH_2_COOH), 5.1–5.4 ppm (m, C***H***_2_ = CH-) and 5.88 ppm (m, CH_2_ = C***H***-) confirmed the successful synthesis of APEG-PAsp (Figure 10b). Finally, 12-mercaptododecanic acid was conjugated to the terminal of APEG-PAsp through the thiol-ene click reaction. The ^1^H-NMR analysis at 1.2 ppm (s, -(C***H***_2_)_9_-), 2.6–2.9 ppm (d, -C***H***_2_COOH), 3.56 ppm (s, -OC***H***_2_C***H***_2_O-) and 4.60 ppm (s, -C***H***CH_2_COOH) confirmed the successful synthesis of APEG-PAsp (Figure 10c).

### 2.3. siRNA-Complexing Behavior of Cationic Copolymer cPEG-CD

**Figure 11:**
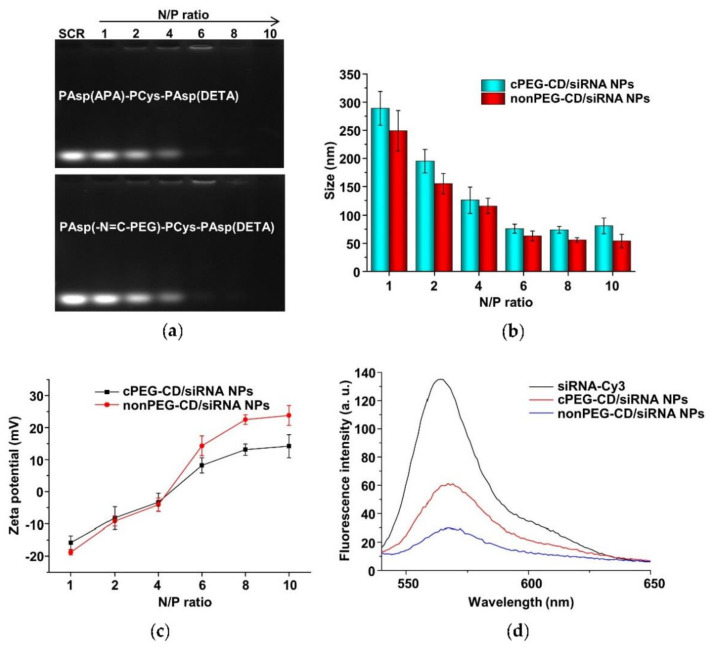
(**a**) Electrophoretic mobility of scrambled siRNA (SCR) in agarose gel after complexation with PAsp(-N=C-PEG)-PCys-PAsp(DETA) and PAsp(APA)-PCys-PAsp(DETA) at various N/P ratios. Particle sizes (**b**) and zeta potentials (**c**) of cPEG-CD/siRNA NPs and nonPEG-CD/siRNA NPs at various N/P ratios and pH 7.4. Data are shown as the mean ± SD (n = 3). (**d**) Fluorescence profiles of siRNA-Cy3, cPEG-CD/siRNA-Cy3 NPs and nonPEG-CD/siRNA-Cy3 NPs.

The cationic copolymers cPEG-CD (i.e., PAsp(-N=C-PEG)-PCys-PAsp(DETA)) with comb-like PEG side chains and nonPEG-CD without PEG side chains (i.e., PAsp(APA)-PCys-PAsp(DETA)) were used in complex with siRNA to determine their complexing behaviors. The nanoparticles (i.e., cPEG-CD/siRNA nanoparticles and nonPEG-CD/siRNA nanoparticles) were prepared at different N/P ratios using the cationic polymer cPEG-CD, or nonPEG-CD, respectively, for complexing with siRNA. Agarose gel retardation analyses were performed to evaluate the siRNA-complexing behaviors. At N/P ratios of above six, the mobilities of siRNA were completely retarded in both cPEG-CD/siRNA nanopartilces (cPEG-CD/siRNA NPs) and nonPEG-CD/siRNA (nonPEG-CD/siRNA nanoparticles (nonPEG-CD/siRNA NPs) (Figure 11a), indicating the complete complexation of siRNA with both cPEG-CD and nonPEG-CD. Moreover, the reason that the band of siRNA was hardly detected at N/P ratios of 8 and 10 may be due to the highly compact structure of polyplexes [40]. In this structure, the siRNA dye GoldenView^TM^ may hardly insert into siRNA, and the aggregation of dye may cause a fluorescence quench effect in the siRNA dye. In addition, the siRNA in the polyplex was hardly degraded because of the protection of cationic polymers [34]. The N/P ratio indicates the moles of nitrogen (N) in the cPEG-CD polymer relative to the moles of phosphate groups (P) in the siRNA. To achieve high transfection efficiency and low cytotoxicity, cationic polymers should complex with siRNA at a critical charge neutralization ratio (i.e., N/P ratio). Above this appropriate N/P ratio, extra polymers contributed to the free polymer population and high cationic cytotoxicity [41]. Here, the amount of cationic polymer cPEG-CD at the N/P ratio of six was enough to condense the siRNA, forming a tight and small polyplex (Figure 11b). Excess cPEG-CD polymers did not significantly decrease polyplex size but may bring about more free cationic polymers, resulting in higher cytotoxicity. Therefore, the N/P ratio of six at pH 7.4 is a suitable ratio for follow-up experiments. Moreover, the zeta potentials of cPEG-CD/siRNA NPs and nonPEG-CD/siRNA NPs showed significant differences at the same N/P ratio, while their sizes were similar. As shown in Figure 11b and c, at an N/P ratio of six, the cPEG-CD/siRNA NPs had a lower zeta potential (8.2 ± 2.4 mV vs. 15.2 ± 3.1 mV) but similar size (76.2 ± 8.1 nm vs. 62.0 ± 9.2 nm) when compared with nonPEG-CD/siRNA ones.

These results indicated that the comb-like PEG side chains limited the complexation of siRNA with polymer cPEG-CD, reducing the siRNA-complexing content and positive charges. When siRNA-Cy3 complexed with polymer to form polyplexes, the fluorescence intensity of siRNA-Cy3 significantly decreased due to the fluorescence quench effect. The higher the siRNA-Cy3 content of any one polyplex, the stronger the fluorescence quench effect that was observed. The cPEG-CD/siRNA-Cy3 NPs showed higher fluorescence intensity (~57) than the nonPEG-CD/siRNA-Cy3 NPs (~27) due to the lower siRNA content and the weaker fluorescence quench effect (Figure 11d), which was consistent with the results of nanoparticle size and zeta potential.

### 2.4. Preparation and Characterization of Small Polyplexes

We supposed that the polyplex sizes may decrease if the comb-like PEG side chains were removed. To confirm this hypothesis, we introduced the pH-sensitive imine bond between PEG side chains and the polyaspartic acid backbone. Moreover, the crosslinking between thiol groups in the PCys blocks could stabilize the polyplexes during the nanoparticle fabrication process and blood circulation.

**Figure 12:**
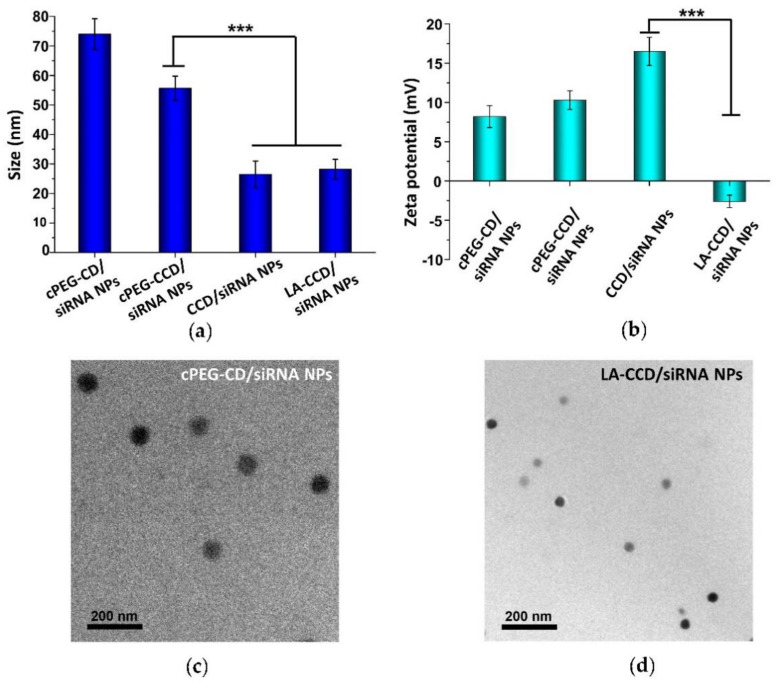
Particle sizes (**a**) and zeta potentials (**b**) of cPEG-CD/siRNA NPs, cPEG-CCD/siRNA NPs, CCD/siRNA NPs, and LA-CCD/siRNA NPs. Data are shown as the mean ± SD (n = 3). *** *p* < 0.001. Transmission electron microscope (TEM) images of cPEG-CD/siRNA NPs (**c**) and LA-CCD/siRNA NPs (**d**).

Next, we prepared and characterized the small polyplexes as follows (Figure 1 and Figure 12): firstly, after cPEG-CD was complexed with siRNA at an N/P ratio of six, the oxygen was bubbled into cPEG-CD/siRNA NPs solution to form a crosslinked interlayer through oxidization crosslinking of the thiol groups, which reduced the size of polyplexes from 76.2 ± 8.1 nm to 55.7 ± 4.1 nm. Then, cPEG-CD/siRNA NPs with crosslinking interlayers were dialyzed against PBS (pH 5.0) to break the imine bond in order to remove the PEG out-layer, which further decreased the polyplex size to 26.5 ± 4.5 nm and increased the zeta potential from + 10.3 ± 1.2 mV to + 16.5 ± 1.8 mV. Finally, the anionic copolymer LA-PEG-PAsp was used to neutralize the positive charge of polyplexes and introduce the targeting ligand lauric acid. After coating with LA-PEG-PAsp, the size of LA/Crosslinked-CD/siRNA nanoparticles (LA-CCD/siRNA NPs) increased slightly to ~28.2 nm and the zeta potential was reduced to -2.6 mV, which was favorable for cell uptake and the prolonging of blood circulation half-life.

Therefore, the pH-sensitive PEG shedding and disulfide bond-crosslinking synergistically reduced the nanoparticle size in the following ways: (i) the crosslinking between thiol groups in the middle block PCys of different polymer strands formed the crosslinked interlayer, which decreased the size of cPEG-CD/siRNA NPs; (ii) the PEG shedding caused by low pH-triggered imide bond breakage further decreased the nanoparticles’ size; (iii) the disulfide bonds-crosslinking interlayer stabilized the nanoparticles after PEG shedding. Besides, the coating with negatively charged LA-PEG-PAsp neutralized the excessive positive charges and introduced the target ligand onto the nanoparticles. Thus, we prepared a crosslinking interlayer-stabilized small nanoparticle with a targeting ligand.

### 2.5. Cellular Uptake and GENE Knock-Down of SMALL Polyplexes

**Figure 13:**
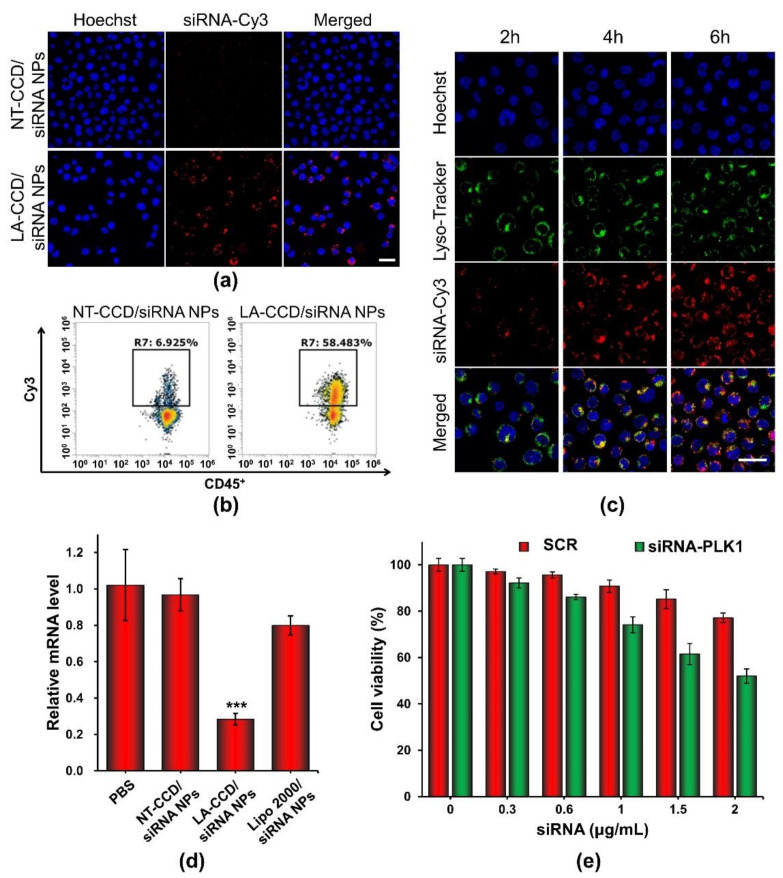
Cellular uptake of NT-CCD/siRNA NPs and LA-CCD/siRNA NPs were observed by (**a**) CLSM and quantified by (**b**) flow cytometry assays. Scale bar, 25 μm. (**c**) Lysosomal escape of LA-CCD/siRNA NPs. Scale bar, 25 μm. The cell nuclei were stained blue with Hoechst 33324, and lysosomes were labeled with the green fluorescence dye LysoTracker™ Green. The relative mRNA level and protein expression of the PLK1 gene in THP-1 incubated with various nanoparticles for 48 h were detected by (**d**) qRT-PCR assay. *** *p* < 0.001. (**e**) Cytotoxicity of LA-CCD/siRNA NPs with various siRNA concentrations to THP-1 cells. Data are shown as the mean ± SD (n = 3).

Flow cytometry and confocal laser scanning microscopy were carried out to evaluate the siRNA delivery efficiency of LA-CCD/siRNA NPs in THP-1 cells. PEG-PAsp was used instead of LA-PEG-PAsp to prepare the polyplexes without a targeting ligand. To monitor the delivery of siRNA, the fluorescence dye Cy3 was used to label siRNA (siRNA-Cy3). As shown in Figure 13a, the neutrally charged polyplex without targeting ligand LA (NT-CCD/siRNA-Cy3 NPs) was hardly internalized by cells because of the poor interaction between NT-CCD/-siRNA-Cy3 NPs and negatively charged cell membranes. However, the neutrally charged polyplex with LA targeting (LA-CCD/siRNA-Cy3 NPs) was effectively internalized by THP-1 cells due to the specific binding between lauric acid and the fatty acid receptor GPR40, which was highly expressed on the THP-1 cell membrane [42,43]. The transfection efficiency of LA-CCD/Cy3-siRNA NPs was quantified using a flow cytometry assay (Figure 13b). The percentages of Cy3 fluorescence positive cells were 58.48% for the LA-CCD/siRNA-Cy3 NPs group and 6.93% for the NT-CCD/siRNA-Cy3 NPs group, respectively. The lysosomal escape capacity of LA-CCD/siRNA-Cy3 NPs was evaluated using a CLSM assay. As shown in Figure 13c, the separation of siRNA-Cy3 and lysotracker fluorescence indicated that the siRNA-Cy3 had escaped from lysosome because of the proton sponge effect of secondary amines in the PAsp (DETA) block. These results demonstrated that the ultra-small polyplexes with the targeting ligand lauric acid could efficiently deliver the siRNA into human leukemic cell THP-1.

Serine/threonine polo-like kinase 1 (PLK1), which is highly expressed in cancer cells, is considered to be one of the cornerstones of the maintenance of normal cell division. Down-regulation of PLK-1 expression by siRNA effectively inhibited tumor growth [44,45]. Thus, we used LA-CCD/siRNA NPs targeting the PLK1 gene to specifically silence PLK1 gene expression. The relative mRNA levels of the PLK1 gene in THP-1 cells treated with different nanoparticles for 48 h were detected by quantitative real-time polymerase chain reaction (qRT-PCR). As shown in Figure 13d, compared with PBS group, the relative mRNA level of the PLK1 gene in the LA-CCD/siRNA NPs group decreased by ~71.59%. Notably, the relative mRNA level of the PLK1 gene in the LA-CCD/siRNA NPs group was significantly lower than that in the LipofectamineTM 2000/siRNA NPs group, while the relative mRNA level of the PLK1 gene in the NT-CCD/siRNA NPs group was similar to that in the PBS group. These results demonstrated that LA-CCD/siRNA NPs had higher inhibition efficiency than the commercial reagent Lipofectamine™ 2000. Subsequently, we evaluated the cytotoxicity of LA-CCD/siRNA NPs incorporating SRC or siRNA-PLK1 to THP-1 cells (Figure 13e). The viability of cells receiving LA-CCD/SCR NPs was above 85% even at a high siRNA concentration of 1.5 μg/mL, suggesting low cytotoxicity of LA-CCD/SCR NPs. On the contrary, the cytotoxicity in the siRNA-PLK1 group was significantly higher than that in the SRC group, suggesting that LA-CCD/siRNA NPs incorporating siRNA-PLK1 efficiently down-regulated PLK1 gene expression and, thereby, inhibited THP-1 tumor cell growth. Above all, the small polyplexes with the targeting ligand lauric acid had low cytotoxicity and efficiently delivered siRNA into THP-1 cells to specifically knock down gene expression.

## 3. Materials and Methods

### 3.1. Materials

4-Benzyl-l-aspartate, 2-hydroxy-4′-(2-hydroxyethoxy)-2-methylpropiophenone, triphosgene and *N*-carboxyanhydride of β-benzyl-l-aspartate (BLA-NCA) were purchased from Aladdin (Shanghai, China). S-tert-butylthio-l-cysteine, α-methoxy-ε-hydroxy-poly(ethylene glycol) (mPEG-OH, *M_n_* = 2 kDa) and 12-mercaptododecanic acid were purchased from Sigma-Aldrich (St. Louis, MO, USA). 4-Dimethylaminopyridine, 4-carboxybenzaldehyde, *N*-hydroxysuccinimide, *N*-(3-dimethylaminopropyl)-*N*’-ethylcarbodiimide hydrochloride, propargylamine, 1-butanamine, diethylenetriamine, tris(2-carboxyethyl)phosphine hydrochloride and 3-azido-1-propanamine were purchased from J&K Chemical Reagent Co. (Beijing, China). α-Allyl-ω-amino PEG (APEG-NH_2_, *M_n_* = 2.4 kDa) was purchased from 3A Chemicals Co. (Shanghai, China). LysoTracker™ Green DND-26, Hoechst 33342 and Cy3-labeled siRNA were purchased from Invitrogen (Gaithersburg, MD, USA). A human leukemic cell line (THP-1) was purchased from the Cell Bank of the Chinese Academy of Science (Shanghai, China) and cultivated in 1640 medium (Gibco, New York, NY, USA) supplemented with 10% fetal bovine serum, and 1% penicillin and streptomycin, under a humidified atmosphere with 5% CO_2_ at 37 °C.

### 3.2. Synthesis of NCA of S-Tertbutylmercapto-L-Cysteine (tBMLC-NCA)

A quantity of 5 g of S-tert-butylmercapto-l-cysteine (23.7 mmol) was suspended in 50 mL ethylacetate. Then, 5 g of triphosgene (16.6 mmol) was dissolved in 30 mL ethylacetate and added dropwise to perform a reaction under refluxing condition. After the reaction solution became clear, the volume of the solution was rotary evaporated to 10 mL. Furthermore, 80 mL petroleum ether was added to obtain white precipitate. Finally, the white precipitate was recrystallized to obtain tBMLC-NCA.

### 3.3. Synthesis of Alkyne-Terminated and IMINE-conjugated PEG (mPEG-C=N-Alkyne)

At first, 2.0 g of mPEG-OH (1 mmol) was vacuum-dried at 70 °C for 2 h in a 50 mL flask and dissolved in 30 mL of anhydrous dichloromethane. Quantities of 1.92 g of EDCI (10 mmol), 1.22 g of DMAP (10 mmol) and 1.5 g of 4-carboxybenzaldehyde (10 mmol) were added into the above solution under N_2_ atmosphere with ice bath cooling. The reaction was kept stirring for 12 h at room temperature. The reaction mixture was dialyzed (MWCO: 1 kDa) against methanol for 1 d, rotary evaporated, and then vacuum-dried to obtain mPEG-CHO. Then, 1.0 g of mPEG-CHO (0.05 mmol) was dissolved in 10 mL of anhydrous dichloromethane in a 25 mL flask. In addition, 319 μL of propargylamine (5 mmol) was added dropwise to the mPEG-CHO solution. The reaction mixture was kept stirring for 48 h at 35 °C. The resulting production was precipitated in excessive cool diethyl ether. The precipitant was washed with diethyl ether, and vacuum-dried to obtain mPEG-C=N-Alkyne.

### 3.4. Synthesis of PBLA

Poly(β-benzyl l-aspartate) (PBLA) was synthesized by ring-opening polymerization of BLA-NCA using n-butylamine as an initiator. In brief, 0.87 g of BLA-NCA (3.5 mmol) was dissolved in 2 mL of anhydrous DMF in a 25 mL flask. Then, 136.8 μL of 1-butanamine (0.35 mmol) dissolved in 10 mL of anhydrous dichloromethane was added into the above solution under N_2_ atmosphere. The reaction was kept stirring for 72 h at 35 °C. The mixture was precipitated in excessive cool diethyl ether. The precipitant was washed with diethyl ether and vacuum-dried to obtain PBLA.

### 3.5. Synthesis of Poly(3-Azidopropylamine) Aspartamide

Poly(3-azidopropylamine) aspartamide, referred to here as PAsp(APA), was synthesized by ammonolysis reaction between PBLA and 3-azidopropylamine. In brief, 0.6 g of PBLA (0.28 mmol) and 5 mL of 3-azidopropylamine (51 mmol) were dissolved in 10 mL of anhydrous DMF, and then the solution was stirred for 6 h at 35 °C. After the reaction, the solution was precipitated in excessive cool diethyl ether. The precipitant was washed with diethyl ether and vacuum-dried to obtain PAsp(APA).

### 3.6. Synthesis of PAsp(APA)-PBMLC-PBLA

PAsp(APA)-PBMLC-PBLA was synthesized using PAsp(APA) as an initiator to, in turn, initiate the ring-opening polymerization of BLA-NCA and tBMLC-NCA. In brief, 0.5 g of Ba-PAsp(APA) (0.24 mmol) was dissolved in 2 mL of anhydrous DMF in a 50 mL flask. Then, 30 mL of anhydrous dichloromethane was added into the above solution. Subsequently, 0.4 g of tBMLC-NCA (1.70 mmol), dissolved in 1 mL of anhydrous DMF, was added into the above solution under N_2_ atmosphere. The reaction was kept stirring for 48 h at 35 °C. After 48 h, 2.73 g of BLA-NCA (10.96 mmol), dissolved in 2 mL of anhydrous DMF, was added into the above solution and the reaction was kept stirring for 48 h at 35 °C. After the reaction, the solution was precipitated in excessive cool diethyl ether. The precipitant was washed with diethyl ether and vacuum-dried to obtain PAsp(APA)-PBMLC-PBLA.

### 3.7. Synthesis of PAsp(-N=C-PEG)-PBMLC-PBLA

Quantities of 0.3 g of PAsp(APA)-PBMLC-PBLA (0.023 mmol), 0.75g of mPEG-C=N-Alkyne (0.345 mmol) and 1.25 mg of CuSO_4_·5H_2_O (0.005 mmol) were dissolved in 10 mL of DMF in a 25 mL flask. The freeze–thaw cycle was run three times to remove oxygen under nitrogen atmosphere, and then 10 mg of sodium ascorbate (0.05 mmol) was added. The reaction was kept stirring for 24 h at 35 °C. After the reaction, 100 μL PMDETA was added to the reaction solution, followed by dialysis (MWCO:14 kDa) against deionic water for 2 d to remove the copper ion and free mPEG-C=N-Alkyne. The purified solution was lyophilized to obtain PAsp(-N=C-PEG)-PBMLC-PBLA.

### 3.8. Synthesis of Triblock Cationic Copolymer with PEG Lateral Chain PAsp(-N=C-PEG)-PCys-PAsp(DETA)

Quantities of 0.5 g of PAsp(-N=C-PEG)-PBMLC-PBLA (0.017 mmol) and 1.65 mL of diethylenetriamine (15.3 mmol) were dissolved in 10 mL of anhydrous DMF, and the solution was stirred for 2 h at 35 °C. Then, 0.24 g of TCEP (0.85 mmol) was added, and the mixture solution was stirred for 6 h at 35 °C in N_2_ atmosphere. After reaction, the solution was dialyzed (MWCO:3.5 kDa) against oxygen-free water for 2 d and lyophilized to obtain PAsp(-N=C-PEG)-PCys-PAsp(DETA).

### 3.9. Synthesis of Triblock Cationic Copolymer without PEG Lateral Chain PAsp(APA)-PCys-PAsp(DETA)

Overall, 0.5 g of PAsp(APA)-PBMLC-PBLA (0.039 mmol) and 3.6 mL of diethylenetriamine (33.3 mmol) were dissolved in 10 mL of anhydrous DMF, and the solution was stirred for 2 h at 35 °C. Then, 0.52 g of TCEP (1.85 mmol) was added, and the mixture solution was stirred for 6 h at 35 °C in N_2_ atmosphere. After the reaction, the solution was dialyzed (MWCO:3.5 kDa) against oxygen-free water for 2 d and lyophilized to obtain PAsp(APA)-PCys-PAsp(DETA).

### 3.10. Synthesis of Anionic Copolymer APEG-PAsp

At first, 0.51 g of APEG-NH_2_ (0.21 mmol, *M_n_* = 2.4 kDa) was vacuum-dried at 70 °C for 2 h in a 100 mL flask, and dissolved in 20 mL of anhydrous dichloromethane. Subsequently, 3.7 g of BLA-NCA (14.9 mmol), dissolved in 4 mL of anhydrous DMF, was added into the above solution under N_2_ atmosphere. The reaction was kept stirring for 48 h at 35 °C. The mixture was precipitated in excessive cool diethyl ether. The precipitant was washed with diethyl ether and dried overnight under vacuum to obtain a white solid. Then, 2 g of APEG-PBLA (0.12 mmol) was dissolved in 10 mL of DMSO in a 50 mL flask. Subsequently, 20 mL of NaOH (1 M) was added dropwise to above solution in ice bath. After the reaction was kept stirring for 3 h at room temperature, the solution was dialyzed (MWCO: 3.5 kDa) against deionized water for 2 d and lyophilized to obtain APEG-PAsp.

### 3.11. Synthesis of Lauric Acid-Terminated Anionic Copolymer LA-PEG-PAsp

A total of 1 g of APEG-PAsp (0.11 mmol) dissolved in 2 mL of deionized water was mixed with 0.51 g of 12-mercaptododecanic acid (2.2 mmol) dissolved in 2 mL of methanol. After adding 20 mg photoinitiator UV2959, the reaction was kept stirring for 1 h under UV light irradiation (RW-UVA 200U, Worun Ltd., Shenzhen, China). The solution was dialyzed (MWCO: 1 kDa) against methanol for 1 d and deionized water for 1 d, and then lyophilized to obtain LA-PEG-PAsp.

### 3.12. Characterizations

^1^H-NMR spectra were recorded on a Bruker 400 MHz spectrometer at room temperature. Fourier transform infrared spectrum (FTIR) measurements were carried out using a Nicolet/Nexus 670 FTIR spectrometer with a resolution of 2 cm^−1^ and in the range between 4000 and 500 cm^−1^. The powder samples were compressed into KBr pellets. The molecular weight distribution of polymers was analyzed using a gel permeation chromatography (GPC) system consisting of a Waters 1515 pump, an Ultrahydrogel^TM^ 500 column, an Ultrahydrogel^TM^ 250 column and a Waters 2417 differential refractive index detector using PEG as a calibration standard. DMF containing LiBr (1 g/L) was used as an eluent at a flow rate of 1.0 mL/min. For transmission electron microscopy (TEM) analysis, 4 μL of the sample solution (0.2 mg/mL) was dropped to the copper grid. After being dried in dryer, the sample was stained with a phosphotungstic acid solution (3 wt%) and observed by a transmission electron microscope (JEOL, Tokyo, Japan) operated at 120 kV. The sizes and zeta potentials of polyplexes were measured by dynamic light scattering (DLS) at 25 °C (Malvern NANO ZS, Malvern Instruments Ltd., Malvern, UK).

### 3.13. Determination of N/P Ratio of Polyplexes

The polymers PAsp(-N=C-PEG)-PCys-PAsp(DETA) and PAsp(APA)-PCys-PAsp(DETA) were complexed with 0.5 μg of siRNA at different N/P ratios, respectively. Polyplexes at different N/P ratios were loaded onto 1% agarose gels with GoldenView (NEB, Beverly, MA, USA). Gels were electrophoresed in 1×TAE buffer at 100 V for 25 min and imaged under UV light in a DNR Bio-Imaging Systems (Jerusalem, Israel) to detect the retardation of siRNA mobility.

### 3.14. Assessment of the Fluorescence Quenching of siRNA-Cy3 Complexed in Polyplexes

The polymers PAsp(-N=C-PEG)-PCys-PAsp(DETA) and PAsp(APA)-PCys-PAsp(DETA) were complexed with 30 μg of siRNA at N/P ratios of six, respectively. The two kinds of polyplexes were abbreviated to cPEG-CD/siRNA NPs and nonPEG-CD/siRNA NPs, respectively. These solutions of cPEG-CD/siRNA NPs, nonPEG-CD/siRNA NPs and free siRNA-Cy3 at a siRNA-Cy3 concentration of 30 μg/mL were measured with a fluorescence spectrometer (Perkin Elmer Ltd., Beaconsfield, UK) to assess the fluorescence quenching of siRNA-Cy3 complexed in polyplexes.

### 3.15. Cellular Uptake of Polyplexes

THP-1 cells were mixed with polyplexes containing siRNA-Cy3 and incubated in 35 mm culture dishes at a density of 1 × 10^6^ cells per dish for 6 h in the dark. Cell nuclei were stained with Hoechst 33342. The cellular uptake of different polyplexes were observed by confocal laser scanning microscope (CLSM, Leica SP8, Leica Microsystems, Wetzlar, Germany). After incubation with different polyplexes for 6 h, THP-1 cells were collected and incubated with CD16/32 blocking antibody for 10 min, then incubated with anti-human CD45-FITC antibody at 4 °C for 30 min. CD45^+^Cy3^+^ THP-1 cells were quantified using a flow cytometer (Attune NxT, Invitrogen, Carlsbad, CA, USA). The data were analyzed by FlowJo software.

### 3.16. Analysis of Lysosomal Escape of Polyplexes and Cytoplasmic Release of siRNA

THP-1 cells were mixed with polyplexes containing siRNA-Cy3 and incubated in 35 mm culture dishes at a density of 1 × 10^6^ cells per dish for various times. Cell nuclei and lysosomes were stained with Hoechst 33342 and LysoTracker™ Green, respectively. The intracellular distribution of siRNA-Cy3 and lysosomes were observed using CLSM.

### 3.17. Quantitative Real-Time Polymerase Chain Reaction (qRT-PCR)

The relative mRNA levels of the PLK1 gene were detected by qRT-PCR. THP-1 cells were mixed with polyplexes containing 1 μg/mL siRNA-PLK1 and incubated in cell culture plates at a density of 1 × 10^6^ cells/mL for 48 h. Then, cells were collected, and the total RNA was extracted using the TRIzol reagent Kit (Invitrogen, Carlsbad, CA, USA), and the first strand cDNA was synthesized using the Prime Script™ RT reagent Kit (Takara, Japan). A qRT-PCR assay was performed using a Fast Start Universal SYBR Green Master (Roche, Basel, Switzerland). The PCR amplification was carried out for 40 cycles under the following conditions: 95 °C for 10 min, 95 °C for 10 s, 60 °C for 30 s and 72 °C for 30 s using the Step One plus Real-time PCR System (ABI, Carlsbad, CA, USA). All experiments were conducted in triplicate. The forward and reverse primer sequences for PLK1 gene were 5′-CTTTTTCGAGGACAACGACTTC-3′ and 5′-GATGAATAACTCGGTTTCGGTG-3′, respectively. The mRNA level of GAPDH gene, a reference gene, was used for normalization of the expression level of mRNA. The forward and reverse primer of GAPDH gene were purchased from Sangon Biotech company (Shanghai, China). The forward and reverse sequences of siRNA-PLK1 were 5′-GAGAAGAUGUCCAUGGAAATT-3′ and 5′-UUUCCAUGGACAUCUUCUCTT-3′, respectively, and were prepared by Sangon Biotech company (Shanghai, China).

### 3.18. Cell Viability

THP-1 cells were mixed with polyplexes containing siRNA-mock (scrambled siRNA as negative control) or siRNA-PLK1 at siRNA concentrations of 0.3, 0.6, 1, 1.5, 2 μg/mL and incubated in 96-well plates at a density of 1 × 10^6^ cells/mL for 24 h. After centrifugation, cell culture medium was replaced with the fresh medium containing 10% FBS and 10 μL MTT solutions (5 mg/mL in PBS) for an additional 4 h at 37 °C. After centrifugation, the culture medium was discarded and 150 μL DMSO was added to dissolve the generated substrate. The ratio of absorbance values at A_570nm_/A_630nm_ for each well were determined using a Tecan Infinite F200 (Crailsheim, Germany). PBS was used instead of polyplexes as the negative control. The cell viability (%) was determined by comparison with the negative control.

### 3.19. Statistics

Data were presented as mean ± standard deviation (SD). Statistical significance was analyzed by two-sided Student’s *t*-test (* *p* < 0.05, ** *p* < 0.01, *** *p* < 0.001; ns, no significance).

## 4. Conclusions

We developed a strategy to engineer the size and surface of polyplexes for siRNA targeting delivery. The multifunctional triblock copolymer endowed the polyplexes with pH-sensitive PEG shedding, disulfide bond-crosslinking, and efficient siRNA complexation, which synergistically decreased the polyplexes’ size. The coating included in this study, with an anionic copolymer modified with a targeting ligand, increased the efficiency of cell-specific siRNA delivery.

## Data Availability

The data presented in this study are available in this article.

## Sample Availability

Not available.
